# The Bacterial Microbiota of Edible Insects *Acheta domesticus* and *Gryllus assimilis* Revealed by High Content Analysis

**DOI:** 10.3390/foods11081073

**Published:** 2022-04-07

**Authors:** Dominykas Aleknavičius, Juliana Lukša, Živilė Strazdaitė-Žielienė, Elena Servienė

**Affiliations:** 1 Laboratory of Genetics, Institute of Botany, Nature Research Centre, 08412 Vilnius, Lithuania; dominykas.aleknavicius@gamtc.lt (D.A.); juliana.luksa@gamtc.lt (J.L.); zivile.strazdaite-zieliene@gamtc.lt (Ž.S.-Ž.); 2 Department of Chemistry and Bioengineering, Vilnius Gediminas Technical University, 10221 Vilnius, Lithuania

**Keywords:** house cricket, Jamaican field cricket, novel food

## Abstract

In the concept of novel food, insects reared under controlled conditions are considered mini livestock. Mass-reared edible insect production is an economically and ecologically beneficial alternative to conventional meat gain. Regarding food safety, insect origin ingredients must comply with food microbial requirements. House crickets (*Acheta domesticus*) and Jamaican field crickets (*Gryllus assimilis*) are preferred insect species that are used commercially as food. In this study, we examined cricket-associated bacterial communities using amplicon-based sequencing of the 16S ribosomal RNA gene region (V3–V4). The high taxonomic richness of the bacterial populations inhabiting both tested cricket species was revealed. According to the analysis of alpha and beta diversity, house crickets and Jamaican field crickets displayed significantly different bacterial communities. Investigation of bacterial amplicon sequence variants (ASVs) diversity revealed cricket species as well as surface and entire body-associated bacterial assemblages. The efficiency of crickets processing and microbial safety were evaluated based on viable bacterial counts and identified bacterial species. Among the microorganisms inhabiting both tested cricket species, the potentially pathogenic bacteria are documented. Some bacteria representing identified genera are inhabitants of the gastrointestinal tract of animals and humans, forming a normal intestinal microflora and performing beneficial probiotic functions. The novel information on the edible insect-associated microbiota will contribute to developing strategies for cricket processing to avoid bacteria-caused risks and reap the benefits.

## 1. Introduction

Over the next three decades, the global human population is expected to reach almost 10 billion people, which, combined with rising welfare, will result in significant resource use and environmental issues [1]. Population and consumption growth continue to drive up food demand and put strain on food supply systems [2,3]. More wildland might be converted to agriculture, allowing more cattle or crops to be raised, hence increasing global food production. However, such a decision would have detrimental consequences for the environment, including increased carbon emissions, high water and land usage, and a rapid loss of biodiversity [4,5]. Therefore, industries are obligated to think of strategies for the sustainable intensification of food production [6].

The suitable options are to consume less meat and/or start growing alternative protein sources of less resource-needy animal origin. Insect mass rearing is one of the solutions suitable for the feed and food industries [7]. Insects do not emit a large amount of waste heat since they are poikilotherms, and as a result, they have a high feed conversion ratio. In comparison to traditional livestock, growing 1 kg of insect mass requires less feed, water, and land area [8]. Insect rearing is also recognized for its economic benefits, as their nutritional values often match or even exceed those of traditional meat [9]. Most cultivating insects produce modest levels of hazardous gases as waste [10] and their manure, known as frass, is considered a good fertilizer [11]. Furthermore, some species are suited for biodegradable waste management, making insects more environmentally friendly and acceptable for circular economy programs [12]. Currently, about 10,000 tons of premium quality insect production is generated per year, while global production and demand are projected to increase fiftyfold over the next decade [13].

Crickets are one of the insect groups suitable for human consumption as food. House cricket (*Acheta domesticus*) is the most popular cricket species [14] and Jamaican field cricket (*Gryllus assimilis*) is an alternative to it [15,16,17]. Crickets have been farmed in Asia, Europe, America, and Africa. The production of crickets worldwide contributes to economic development and livelihood improvement, has a positive effect on climate change, and properly deals with sustainable environment and agriculture [3,7]. Developing countries are leading with the rearing and consumption of crickets, which help to meet food demand; however, the shortage of legal regulations and experienced food insecurity are the main problems [18]. In addition to global concerns, for industrialized Western countries, cricket-based novel food may diminish consumption of meat and have a positive effect on health problems caused by obesity. The production of crickets as food and feed in industrialized countries is subjected to strict regulations [3,18,19]. In Europe, crickets or their products may be used as a food for human consumption after a safety assessment by the European Food Safety Authority (EFSA) and following the European Commission’s (EC) regulations on specific hygiene rules and insects feeding [18,19,20]. Various food safety criteria, such as addressing biological and chemical hazards, must be investigated to eliminate existing risks [19,20,21]. Now, edible insects must meet traditional meat microbiological safety requirements. However, in most cases, it is difficult to equally compare the microbiological loads of whole-body insects and a clean slice of traditional meat. Therefore, colony-forming units (CFU) of microorganisms on fresh raw crickets overreach traditional raw meat requirements [21]. Insect-related bacteria could be important symbionts determining better performance of the host. Additionally, there are other groups of bacterial contaminants that cause insect health problems [22]. Certainly, insects can be vectors that carry species of bacteria dangerous to humans, such as *Bacillus cereus*, *Campylobacter*, *Clostridium*, *Salmonella*, *Listeria*, etc. [19,20]. Harmful bacteria can be linked to the life cycle of insects, acquired from the feeding substrate, or dedicated to insect processing [19,20,21]. Therefore, EFSA suggests for the definition of insect-associated microbial hazards to evaluate both intrinsically associated microorganisms and those introduced during farming or insect processing [20,22].

The bacteria count on mass-reared raw insects varies greatly not only between insect species or different rearers, but also fluctuates between rearing batches from a single rearing company. These findings show that differences in microbial counts can change even if rearing conditions remain the same [23]. Regarding that, the microbial communities and total aerobic counts (TAC) of crickets reared under controlled conditions change even more if they were fed on different substrates [24]. However, microbial contamination can be reduced by incorporating some antibacterial properties-possessing plant-based ingredients in the insect feed formulation [25]. Another option for reducing cricket microbial load is intestinal emptying by starvation before killing and processing them further. However, the effectiveness of this insect harvesting step is debatable. Although the number of Enterobacteriaceae decreases over time, the TAC remains constant and could increase during long periods of cricket starvation [26]. Thermal processing can further reduce the insect bacterial count by blanching (quick thermal treatment), boiling, or freezing (at −18 °C or lower temperature) [27,28]. Compared to nonthermal methods such as mechanical disruption or asphyxiation, freezing reduces TAC slightly. Blanching appears to be a more effective method of reducing microbial contamination [29]. Cricket boiling for a few minutes can reduce the microbial loads to a level suitable for minced meat requirements [28], while 30 min of boiling decreases bacterial counts below the detection limit [30]. Drying is the final step in the insect production process and is used to remove moisture and extend the shelf life of the finished product. Different drying technologies (sun drying, smoke drying, roasting, freeze drying, oven drying, microwaving) have different levels of effectiveness and influence the quality of products (such as sensory characteristics, bioactive compounds and protein extraction efficiency, microbiological safety, and shelf life) [31,32,33]. Different numbers of mesophilic aerobes, Enterobacteriaceae, and lactic acid bacteria are observed on crickets depending on the processing of insects [5,33]. The viable counts of bacteria on fresh *G. assimilis* were reported to reach 7.3 log CFU/g [34]. After crickets were blanched and following oven drying, it was about 6.52 log CFU/g [17]. There are many more scientific data on microbial contamination of house crickets. The count of bacteria in raw *A. domesticus* usually varies between 6.3 and 9.24 log CFU/g, while blanching reduces it to 2.3–4.39 log CFU/g. After drying, bacterial viable counts reach 1–4.8 log CFU/g values [5,23,24,26,27,31,33,34,35,36].

Unfortunately, culture-dependent food microbiome investigations have limited contribution to knowledge. Standard approaches cannot culture more than 99 percent of naturally occurring microorganisms [37]. Moreover, research applying non-cultural methods such as metagenomics analysis can disclose cultivable as well as unculturable bacterial diversity. Pyrosequencing analysis of the 16S rRNA gene revealed that ready-to-be-consumed house crickets possess a high diversity of bacterial operational taxonomic units (OTUs) [5]. Processed in whole and powdered crickets were dominated by three bacterial phyla ascribed to Proteobacteria, Firmicutes, and Bacteroidetes. In sum, these bacterial groups represented up to 98.8 percent of the total bacterial diversity [5]. The same dominant bacteria phyla were established in fresh house crickets using MySeq Illumina [24,38], and were confirmed again in ready-to-use crickets [39,40]. Few studies focusing on the house cricket-associated bacterial microbiota have been published thus far [5,24,38,39,40] but no study exploring the microbial diversity of Jamaican field crickets using a metagenomic approach has been reported.

In this context, the aims of this study were (i.) to provide an in-depth characterization of bacterial communities associated with the house cricket (*Acheta domesticus*) and Jamaican field cricket (*Gryllus assimilis*) by using the Next-Generation Sequencing (NGS) approach; (ii.) to perform a comparative analysis of bacterial populations associated to the surface and whole body of crickets by uncovering potentially beneficial and pathogenic microorganisms; (iii.) to assess the microbial contamination of processed house crickets and Jamaican field crickets. Scientific studies on the microbiological safety of edible crickets need to be carried out for markets and consumers; therefore, the obtained knowledge will contribute not only to the elucidation of the edible insect-associated microbiota, but also for the formulation of the most efficient raw cricket production steps and the setting of conditions to avoid bacterial risk.

## 2. Materials and Methods

### 2.1. Crickets

Experimental house crickets (*Acheta domesticus*) and Jamaican field crickets (*Gryllus assimilis*) were gained from colonies maintained in the Nature Research Centre, Vilnius, Lithuania. Climate chamber conditions were set at 27 ± 2 °C, 12:12 h of the day:night light cycle, and 40–50% relative humidity. Both species were kept separately in 130 L plastic bins covered by lids with an aluminum mesh. Egg cartons were used as space extenders for crickets. They were vertically stacked side by side to one another through the entire space of the box. Water for the insects was added into a 3 L sealed plastic container placed on top of egg cartons with wicks sticking out of it. Crickets were able to drink out of a wet wick and had no access to open water so as not to spoil it. Feed was placed on top of egg cartons in aluminum plates (33.3 × 23.3 cm). As cricket feed, quail compound feed was used (Dobele, Latvia). According to the manufacturer, most of the feed ingredients are corn, wheat, soybean meal, sunflower meal, and rapeseed oil. There are added vitamins and microelements. Total crude protein content was 20.25%, crude fat 5.26%, crude fiber 13.17%, calcium 3.4%, phosphorus 0.81%, sodium 0.16%, ash 13.17%, lysin 1.25%, and methionine 0.65%. In terms of composition and nutritional specification, this feed is similar to the ones specifically dedicated to the cultivation of crickets [41,42]. The feeding substrate was refiled periodically according to the need. When the crickets became adults, a box with wet coconut husks was placed in the bin. The husks were heat-treated by soaking them in boiling water and used after cooling to room temperature. This substrate was dedicated to collect eggs after crickets oviposited in it. Crickets were allowed to lay eggs for approximately 3–4 days. After this period, the box with the eggs was placed into a newly prepared growing bin.

For the experiments, randomly selected 45–55-day old adult crickets were separated from the maintained population. Live insects were immobilized by squeezing their heads and used for bacteria sampling.

### 2.2. Sample Preparation

#### 2.2.1. Sampling of Microorganisms from the Surface of Crickets

Freshly immobilized crickets (400 g) were mixed with 800 mL of sterile TE buffer (10 mM Tris-HCl, pH 8.0, 1 mM EDTA) and incubated at 20 °C for 45 min with orbital shaking at 120 rpm. The outwashes were filtered through a 1.5 mm wired mesh and the supernatant was centrifuged in 50 mL Falcon test tubes at 5000 rpm for 20 min. The obtained pellet was collected in Eppendorf tubes, centrifuged at 12,000 rpm for 10 min, and used for microbial DNA extraction.

#### 2.2.2. Sampling of Microorganisms from the Whole-Body of Crickets

Thirty grams of washed crickets were aseptically homogenized for 3 min with sterile mortar and pestle in 50 mL of TE buffer. The remnants of the crickets were removed by filtering the homogenate through 1.5 mm wired mesh and following centrifugation at 800 rpm for 10 min. The supernatant was transferred into new tubes and the pellet was collected by centrifugation at 12,000 rpm for 10 min. The pellet was stored at –20 °C and used for the extraction of microbial DNA.

### 2.3. DNA Extraction

The microbial DNA was isolated from sediments obtained from whole-body homogenized crickets and surface-associated samples (about 50 mg) using the manufacturer’s protocol for the Genomic DNA purification kit (Thermo Fisher Scientific Baltics, Vilnius, Lithuania). The quality and quantity parameters of the extracted DNA were assessed by optical reading at 260, 280, and 234 nm, using NanoPhotometer P330 (Implen GmbH, Munich, Germany).

### 2.4. Amplicon Sequencing

The extracted DNA was used to study bacterial diversity by targeting hypervariable regions V3 and V4 of the 16S rRNA gene for sequencing, using 341F/785R primers [43]. Targeted amplicon libraries were generated using Illumina adapters (www.illumina.com, accessed on 6 October 2021), verified on an Agilent Technologies Bioanalizer DNA 1000 and sequenced in pair-end mode (2 × 300) on an Illumina MiSeq platform (Macrogene Inc., Seoul, Korea). All sequences obtained during this work are available in the Sequence Read Archive (SRA) of the National Center for Biotechnology Information (NCBI), under accession number PRJNA806726.

### 2.5. Processing and Analysis of the Sequencing Data

Macrogen provided demultiplexed sequence data in FASTQ format, which were imported for processing into the QIIME2 v2020.06 edition of the QIIME program [44]. In essence, amplicon primers were first deleted with the Cutadapt 2.8 [45]. The DADA2 plugin was used to denoise, filter, and trim the reads (where the median quality rating decreased below 30) [46]. The Greengenes v13_5 database [47] was used to classify amplicon sequence variants (ASVs) in QIIME2, with a classifier trained on the amplified region [48]. For the dataset, the majority taxonomy with seven levels was employed, i.e., this taxonomy was taken to species level, but because species-level identification was not full, we chose to utilize genus-level classifications. Log10 read counts and the phylogeny align-to-tree-mafft-fastree (MAFFT multiple sequence alignment program) [49] plugin in QIIME2 were used to generate a de novo phylogenetic tree utilized in downstream assessments of diversity that included phylogenetic distances. QIIME2 generated stacked bar graphs representing the relative abundance of % distinct species in samples.

Shannon’s Diversity, Faith’s Phylogenetic Diversity (PD), and Pilou’s Evenness indexes were calculated per sample within QIIME2 using rarefied counts to determine alpha (within one sample) diversity (i.e., subsampled to the same sequencing depth across samples; rarefied to 40,000). Excel 2019 was used to construct boxplot figures for alpha diversity. Beta (between-samples) diversity was calculated using the Bray–Curtis dissimilarity statistic [50] based on compositional dissimilarity between samples taking abundance into account, unweighted UniFrac distances that measure phylogenetic distances between taxa, or weighted UniFrac distances that measure phylogenetic distances, and additionally accounting for relative abundance. Principal coordinates (PCoA) plots were created in QIIME2 using the EMPeror graphics tools [51]. Between-group statistical differences were established using weighted and unweighted UniFrac distance metrics and permutational analysis of variance (PERMANOVA, 999 permutations). The ggplot2 package in R was used to create the heatmap [52].

### 2.6. Processing of Crickets

Jamaican field crickets and house crickets before microbiological analysis underwent several processing steps, such as rinsing, boiling, and drying. For rinsing, raw crickets were placed on 1.5 mm wired mesh and washed with sterile deionized running water for 1 min. For thermal processing, washed crickets were placed in boiling water for 5 min. The final processing step samples were prepared by washing, boiling, and oven drying crickets for 13 h at 75 °C. After treatment, the samples were placed in sterile glass flasks covered with aluminum foil and soon after used in the following microbial analysis experiment.

### 2.7. Microbial Analysis of Crickets and Feeding Substrate

Microorganisms of raw and processed crickets were sampled as described in Section 2.2.2.

For the analysis of quail compound feed microbial contamination, 30 g of feeding substrate were washed with 50 mL of sterile 0.9% NaCl solution. The feeding substrate residue was removed by centrifugation of the outwashes at 800 rpm for 10 min. The supernatant was transferred into a new tube, serially diluted, and applied for the analysis of microbial loads.

For the evaluation of total aerobic counts (TAC), serially diluted in sterile 0.9% NaCl solution aliquots were spread onto standard plate count agar (PCA) (0.5% tryptone, 0.25% yeast extract, 0.1% glucose, 1.5% agar) plates followed by incubation at 30 °C for 48 h. After incubation, the colonies were counted as colony-forming units (CFU), then the logarithmic transformation and the mean value of log CFU per gram of crickets or feeding substrate was calculated. Microbiological counts were carried out in triplicates. One-way analysis of variance (ANOVA) was used to compare TAC on fresh and processed crickets. The *p*-value of <0.05 was considered statistically significant.

### 2.8. Viable Bacteria Identification

Randomly selected colonies were used for molecular identification. For identification of bacteria, the V3–V4 region of the 16S rRNA gene was amplified with primers W001 5′AGAGTTTGATCMTGGCTC3′ and W002 5′GNTACCTTGTTACGACTT3′. The PCR was performed directly from the bacterial suspension without DNA extraction. The reaction mixture consisted of 5 µL DreamTaq buffer, 1 µL of 2 mM dNTP mix, 1 µL of each primer (10 µmol/L), 2.5 units of DreamTaq DNA polymerase (Thermo Fisher Scientific Baltics, Vilnius, Lithuania), 1 µL of bacterial suspension in PCA medium, and sterile distilled water up to 50 µL. The following PCR conditions were used: an initial denaturation at 95 °C for 5 min, followed by 30 cycles of 94 °C for 30s, 45 °C for 30 s, and 72 °C for 2 min. The final extension was carried out at 72 °C for 10 min. PCR products were purified using GeneJet PCR purification kit (Thermo Fisher Scientific Baltics, Vilnius, Lithuania) and sequenced using W001 and/or W002 primers at BaseClear (Leiden, The Netherlands). The generated sequences were compared with those found in the FASTA network service of the EMBL-EBI database (http://www.ebi.ac.uk/Tools/sss/fasta/nucleotide.html (accessed on 30 March 2022).

## 3. Results

### 3.1. Diversity and Richness of Acheta domesticus and Gryllus assimilis Bacterial Communities

The bacterial community of Jamaican and house crickets raised under controlled conditions was revealed by Next-Generation Sequencing of V3–V4 region of 16S rDNA amplified from total DNA extracted from surface and whole-body of both cricket’s species. The four variations (JS—Jamaican cricket surface; JW—Jamaican cricket whole-body; HS—house cricket surface; HW—house cricket whole-body) had three biological replicates each. A total of 2.36 million raw paired-end reads were generated across 12 samples. The number of reads ranged from 179,088 to 224,948, with an average of 196,223 per sample (Table 1).

After the pre-processing and quality filtering of reads, a total of 601,809 high quality reads were recovered with an average of 50,150 sequences per sample (Table 1). The number of joined paired-end reads were comparable in whole-body or surface-associated *Acheta domesticus* and *Gryllus assimilis* samples. Rarefaction plots based on Shannon’s diversity index demonstrated that maximum alpha diversity is achieved at 7000 reads and confirmed equivalent alpha diversity in the range of read depths from 7000 to more than 35,000 (Figure S1).

The clustering of the sequences at 97% sequence identity generated a total of 2527 amplicon sequence variants (ASVs). The total number of ASVs detected in individual samples ranged from 190 to 231. Based on analysis of prokaryotic sequences, the lowest number of ASVs was observed in Jamaican cricket whole-body samples 611 (208 ± 12.1, hereafter median for 3 samples ± standard deviation) and house cricket surface samples 614 (204 ± 8.02). The richness of the ASVs was slightly higher in Jamaican surface samples (636 (205 ± 12.12)) and whole-body house cricket samples (666 (223 ± 9.54)) (Table 1).

**Figure 1:**
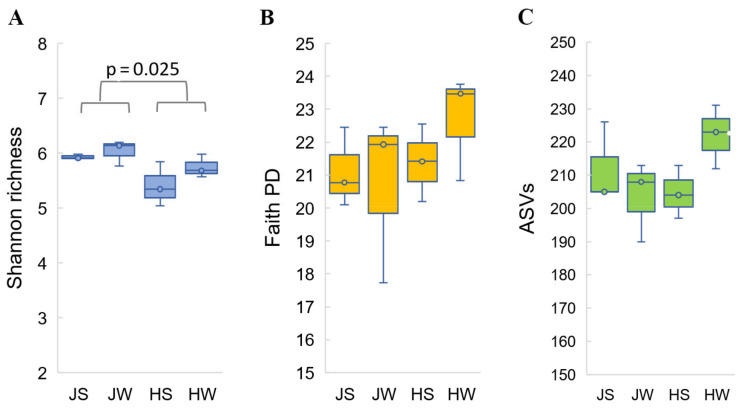
Alpha diversity analysis of Jamaican and house cricket’s bacterial microbiota based on Shannon index (**A**), phylogenetic diversity Faith’s PD index (**B**), and observed ASV index (**C**). Samples were rarefied to sampling depths of 40,000. Kruskal–Wallis test was performed to analyze statistical significance.

Sequencing depth was comprehensive enough to estimate microbial diversity in all single samples. Alpha diversity metrics, such as Shannon diversity index, observed ASVs index and Phylogenetic diversity index (Faith’s PD), did not reveal statistically significant differences in bacterial diversity among the four testing groups (external and whole-body samples of both cricket species) (Figure 1). These estimates match the results of a pseudo-F statistical analysis of both weighted and unweighted sample groups using permutational multivariate analysis of variance (Permanova). Samples JS, JW, HS, and HW did not show statistically significant differences in beta diversity (*p* > 0.05) (Table 2.).

However, when external and whole-body samples were combined, bacterial diversity became apparent between cricket species themselves. Based on the alpha diversity analysis performed with the nonparametric Kruskal–Wallis test and the beta diversity analysis performed on weighted and unweighted UniFrac distance metrics, statistically significant differences between Jamaican vs. house crickets were observed (Shannon diversity, *p* = 0.025; UniFrac unweighted, *p* = 0.004 and weighted, *p* = 0.018) (Figure 1, Table 2).

### 3.2. Bacterial Community Profiling of Jamaican and House Crickets

**Figure 2:**
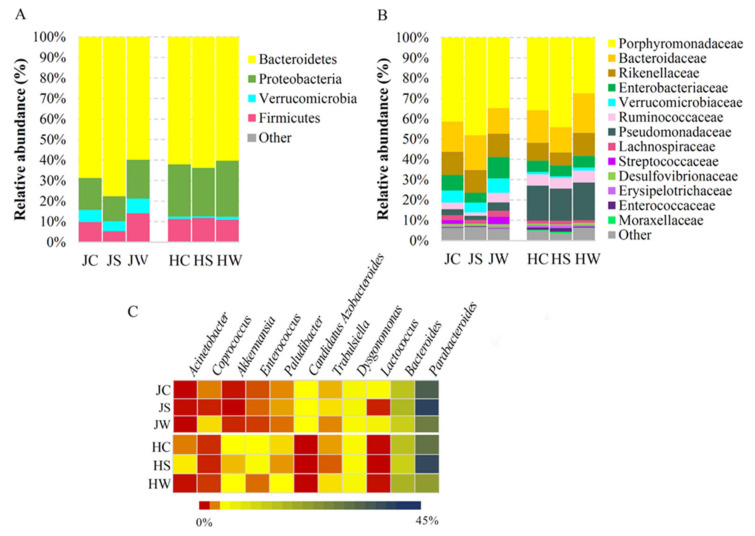
Taxonomic composition of Jamaican and house cricket’s microbiota. Relative abundance of ASVs at phylum (**A**) and family (**B**) level. Heatmap (**C**) demonstrates most abundant ASVs at genus level. JS—Jamaican cricket surface, JW—Jamaican cricket whole-body, JC—combined surface and whole-body samples of Jamaican crickets. HS—house cricket surface, HW—house cricket whole-body, HC—combined surface and whole-body samples of house crickets.

The bacterial microbiota associated with Jamaican and house crickets showed slight differences at the highest taxonomic level. Both cricket species carried bacterial DNA sequences assigned to the four main phyla, collectively accounting for more than 99% of the total bacterial population (Figure 2A, Table S1). The most prevalent phylum on both crickets was Bacteroidetes (68.79% on Jamaican crickets and 62.09% on house crickets), which was mainly represented by Bacteroidia prokaryotic microorganisms at class level (Table S1, Figure S2). The phylum of Proteobacteria was dominant in house crickets compared to Jamaican (25.43% and 15.50%, respectively), while the phylum of Verrucomicrobia was more abundant in Jamaican crickets (Jamaican vs. house crickets, 5.94% and 1.20%, respectively). The majority of microorganisms in the Proteobacteria phylum at the class level were assigned to Gammaproteobacteria and they prevailed inside the crickets (Figure 2A, Table S1). The microorganisms from the Firmicutes phylum were observed at a comparable level on both cricket species (JC—9.45%, HC—10.91%), with slightly increased abundance in whole-body Jamaican cricket samples (JW—13.76%).

Metagenomic analysis of Jamaican and house crickets reared under controlled conditions revealed differences in the composition of the bacterial community at a lower taxonomic level. In total, 35 families and 35 genera were differentiated during this study. The members of the Porphyromonadaceae family constituted the major bacterial group of both cricket species (41.37% for JC and 35.79% for HC), followed by Bacteroidaceae (14.92% for JC and 16.04% for HC) and Rikenellaceae (11.34% for JC and 8.79% for HC) (Figure 2B). Porphyromonadaceae was represented by bacteria of Parabacteroides, *Dysgomonas* and *Paludibacter* genera (Figure 2C). *Parabacteroides* was the most dominant taxon at genus level (36.17% for JC and 31.97% for HC), with higher prevalence on the surface of tested insects (42.57% for JS and 41.09% for HS) (Figure 2C). The Bacteroidaceae family was represented by members of *Bacteroides* genus distributed similarly on both cricket species (14.32% for JC and 14.65% for HC) (Figure 2C). ASVs assigned to *Parabacteroides* and *Bacteroides* genera formed the core microbiomes in HC and JC samples (Table S1). The abundance of bacteria belonging to the Pseudomonadaceae family was more than five-fold higher in house crickets vs. Jamaican field crickets (17.21% for HC and 3.05% for JC). In contrast, the Verrucomicrobiaceae family was significantly more represented in Jamaican field crickets (5.94% for JC and 1.20% for HC). Distributed in a low frequency, the *Lactococcus*, *Candidatus Azobacteroides*, and *Coprococcus* genera were more prevalent in Jamaican field crickets (1.95%, 1.28%, and 0.5%, respectively). Meanwhile, *Enterococcus*, *Akkermansia*, and *Acinetobacter* genera were more represented in the house cricket (1.16%, 1.03%, and 0.49%, respectively) (Figure 2C).

### 3.3. Comparison of Jamaican Field Cricket and House Cricket Bacterial Communities

**Figure 3:**
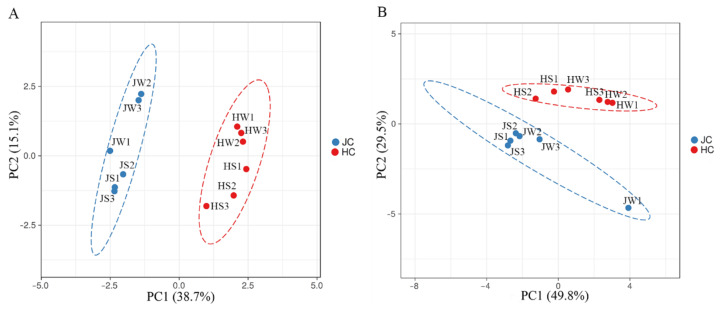
Comparison of bacterial microbiota on Jamaican and house crickets by principal coordinate analysis (PCoA). Plots were calculated using weighted (**A**) and unweighted (**B**) UniFrac distances. JS—Jamaican cricket surface, JW—Jamaican cricket whole-body, HS—house cricket surface, HW—house cricket whole-body. Each dot represents a distinct sample.

Principal coordinate analysis (PCoA) based on both weighted and unweighted UniFrac distances showed clear separation of Jamaican and house cricket samples, thus pointing to differences in the bacterial microbiota composition (Figure 3).

**Figure 4:**
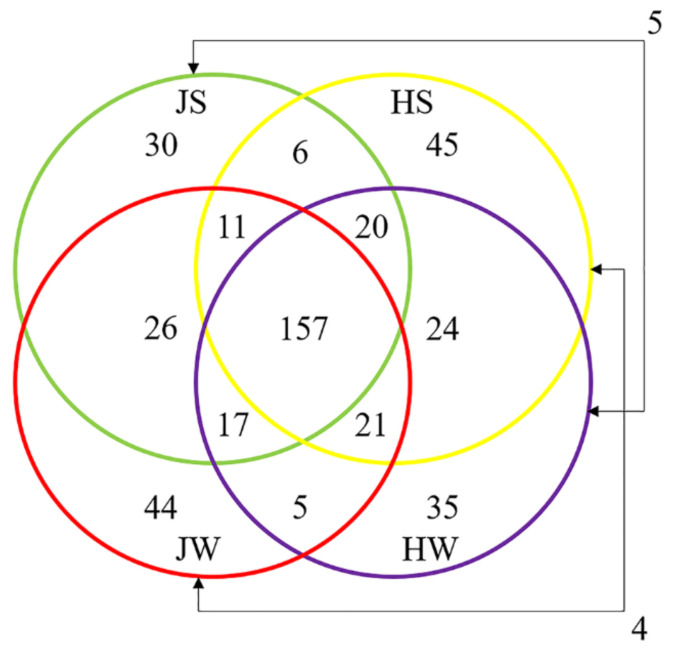
Venn diagram illustrating the number of unique and shared ASVs among Jamaican field and house cricket samples. JS—Jamaican cricket surface, JW—Jamaican cricket whole-body, HS—house cricket surface, HW—house cricket whole-body.

The distribution of unique ASVs between sample groups is illustrated by a Venn diagram (Figure 4). A total of 450 unique ASVs identified in this study, 100 were exclusive to Jamaican field crickets and 104 to house crickets, while 237 ASVs were shared by both cricket species. By comparing surface-associated ASVs, 194 were common to both cricket species, while 78 ASVs were distributed only on JS and 94 on HS samples. The distribution of ASVs in the whole body was similar to surface samples; 200 ASVs were shared by both HW and JW, while 85 ASVs were unique to JW and 84 to HW.

**Figure 5:**
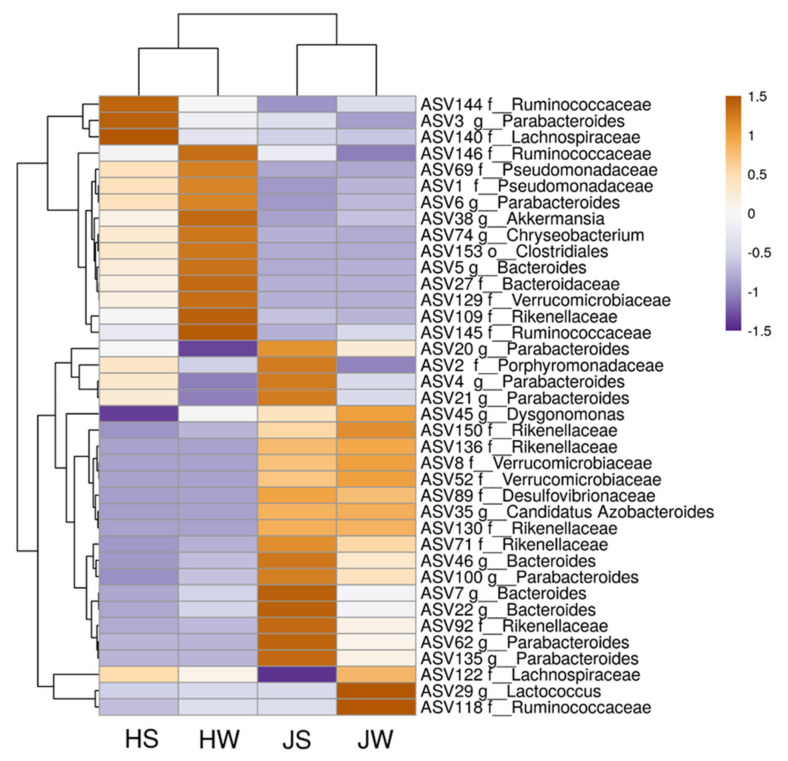
Heatmap of the most abundant unique bacterial ASVs on the Jamaican field and house cricket surface and whole-body samples. JS—Jamaican cricket surface, JW—Jamaican cricket whole-body, HS—house cricket surface, HW—house cricket whole-body.

The heatmap depicts the distribution of the most common ASVs (Figure 5). Based on hierarchical cluster analysis, the structure of microbial community differs in Jamaican field crickets and house crickets.

Among bacterial community, ASVs of Pseudomonadaceae (ASV1, ASV69) and *Akkermansia* (ASV38) inhabited mainly house crickets, while those belonging to Verrucomicrobiaceae (ASV8, ASV52), *Lactococcus* (ASV29), and *Candidatus Azobacteroides* (ASV35) were more abundant on Jamaican field crickets. Among the most abundant *Parabacteroides* genera, a different distribution of closely related microorganisms was observed in both crickets: ASV3 and ASV6 were present mainly in house cricket samples, while ASV4, ASV21, and ASV62 dominated on Jamaican crickets. A similar distribution pattern was observed with *Bacteroides* genera: ASV5 was more abundant in house crickets, while ASV7 and ASV22 were present in Jamaican field cricket samples. Looking at the surface and whole-body samples, differences in ASVs distribution were also visible. A higher abundance of ASVs matching to *Parabacteroides* (ASV3, ASV4, ASV21, and ASV62) and Porphyromonadaceae (ASV2) was documented on the surface of Jamaican or house crickets as compared to whole-body samples. In contrast, some ASVs, such as *Akkermansia* (ASV38) and *Lactococcus* (ASV29), dominated in the interior of crickets.

### 3.4. Microbial Analysis of Crickets and Feeding Substrate

**Figure 6:**
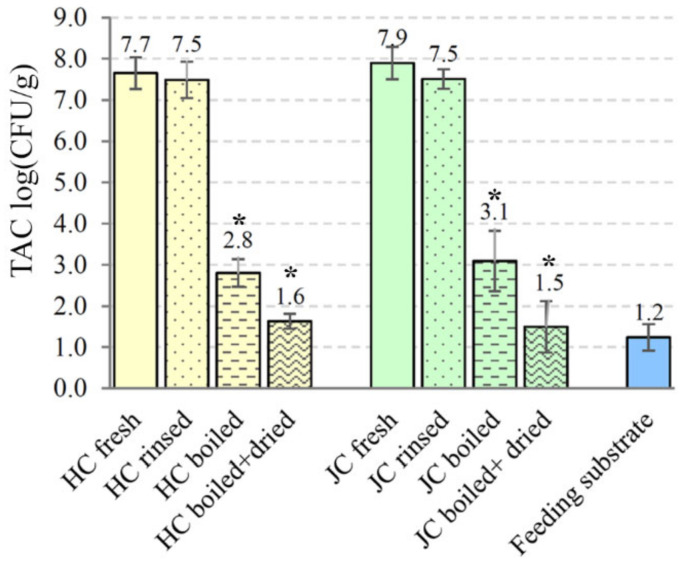
Distribution of the total aerobic plate counts (TAC, log CFU/g) for differently processed *A. domesticus* and *G. assimilis* crickets and feeding substrate. HC—house cricket, JC—Jamaican field cricket. All values are the mean of 3 replicates with ± standard deviation (SD). Asterisk above the column indicates statistically significant differences (*p* < 0.05) between fresh and processed crickets.

The microbiological safety aspects of house crickets and Jamaican field crickets were also analyzed based on bacterial loads of freshly collected and processed crickets. The mean of TAC determined in raw material of *A. domesticus* and *G. assimilis* was comparable—7.65 and 7.90 log CFU/g, respectively (Figure 6). The application of the rinsing step only slightly reduced microbial loads. The level of total viable counts for rinsed house crickets was 7.50 ± 0.44 log CFU/g and 7.51 ± 0.24 log CFU/g for Jamaican field crickets. A statistically significant reduction in microbial counts was observed after thermal processing of both cricket species. A reduction of about 4.85 log CFU/g (*p* = 0.00008) was detected after boiling *A. domesticus* in a kettle of water for 5 min. The introduction of a drying step decreased total bacterial counts to 1.63 ± 0.18 log CFU/g (*p* = 0.00002). Similar findings were observed in the case of *G. assimilis*: 3.09 ± 0.73 and 1.49 ± 0.62 log CFU/g of total aerobic counts were recovered from boiled and additionally dried crickets, respectively. The TAC levels in both processing steps decreased significantly (*p* ˂ 0.0006) comparing to unprocessed *G. assimilis* crickets. Since cricket feeding substrate could be an important source of microbial contamination, quail compound feed was analyzed for microbiological quality. The TAC level observed in the cricket feeding substrate was low—1.24 ± 0.32 log CFU/g of material.

Based on molecular identification of isolated bacteria, representatives of *Bacillus*, *Staphylococcus*, *Micrococcus*, and *Pseudomonas* were mainly observed on tested crickets or feeding substrate (Table S2). In some samples, bacteria from *Acinetobacter*, *Moraxella*, *Enterococcus*, or *Rhodococcus* genera were identified. Greater diversity of isolated bacteria was detected on unprocessed crickets. Even though the total number of bacteria decreased during the processing of crickets, certain bacterial species, observed in raw material or even cricket feeding substrate, remained in boiled and dried crickets. Among those bacteria are *Staphylococcus epidermidis*, *Micrococcus luteus*, *Staphylococcus warneri*, and *Bacillus subtilis* species. Most likely, such heat-resistant bacteria were not eliminated completely using cricket processing.

## 4. Discussion

Numerous insect species from Orthoptera, Hymenoptera, Coleoptera, etc. orders are consumed worldwide at different stages of the development [5]. Living and processed edible insects through transferred microorganisms or bioactive compounds can affect the health of consumers (humans and animals). Therefore, the microbial communities associated with edible insects need to be evaluated by paying attention to potentially beneficial and pathogenic bacteria.

The bacterial characterization of cricket species *Acheta domesticus* and *Gryllus assimilis* was carried out through a Next-Generation Sequencing analysis. This study allowed for an in-depth evaluation of the cricket-associated bacterial communities. The number of high-quality reads recovered from either the whole body or surface of Jamaican field and house crickets was comparable to those resolved by others on Illumina MiSeq platform powdered house cricket samples [39]. When 16S rRNA amplicon pyrosequencing was applied on house crickets, the efficiency of the reads obtained was more than tenfold lower [5]. This could be due to differences in the processing of insects, DNA preparation, and sequencing strategies. The richness of bacterial community on the surface and in the whole body of both cricket species was higher compared to previous studies performed by others. The number of OTUs detected previously in fresh house cricket samples was lower compared to our study (the number of OTUs ranged from 313 to 402) [24,38]. In processed house cricket samples, the variety of OTUs decreased even more to 157 and 175 [5].

Alpha and beta diversity analysis revealed statistically significant differences between entire bacterial communities associated with Jamaican field and house crickets. However, when individual samples, such as surface and whole-body, were examined, the differences became insignificant. House cricket bacterial diversity has previously been shown to differ between rearing companies and there is only a slight difference between the same company rearing production cycles [38]. All these findings are not surprising, because insect bacterial diversity can fluctuate due to variations in living space, feed, and insect species themselves [53,54]. The latter was demonstrated in the Jamaican and house cricket case in the present study.

The structure of bacterial community associated to Jamaican field and house crickets showed slight differences at the highest taxonomy level. Four main bacterial phyla were observed, with a higher abundance of Bacteroidetes on both crickets tested. However, Proteobacteria dominated in house crickets, while Verrucomicrobia was more abundant in Jamaican field crickets. Previous metagenomic studies have revealed the high abundance of three bacterial phyla Bacteroidetes, Proteobacteria, and Firmicutes in fresh and processed house crickets. There was only a difference in ratio between them. Similar to our finding, Bacteroidetes dominated in almost all crickets studied [5,24,38,39,40]. Starting from the family taxonomic level, the differences in the composition of Jamaican and house cricket bacterial community became more obvious. The core microbiomes forming *Parabacteroides* and *Bacteroides* genera dominated on both cricket species. Significant amounts of bacteria from these genera are also found in both fresh [38] and processed house crickets [5,39,40]. *Parabacteroides* and *Bacteroides* have also been observed in cricket feed, suggesting possible spreading through the food chain [55]. The abovementioned microorganisms are one of the most common bacteria in the human body, they populate the mouth, upper respiratory tract, urogenital tract, and, most notably, the intestinal tract of humans and other animals [56]. Representatives of these genera can resist intestinal inflammation, suppress the growth of pathogens, and accelerate the establishment of intestinal microbial balance [57]. Nevertheless, some species can act as opportunistic pathogens, causing infections in immunosuppressed hosts [56]. Differences in the microbial communities of Jamaican field and house crickets were mainly caused by the genera distributed at a low frequency. Representatives of the genera *Lactococcus*, *Candidatus Azobacteroides*, and *Coprococcus* were more prevalent in Jamaican field crickets, while *Enterococcus*, *Akkermansia*, and *Acinetobacter* more inhabited house crickets. It should be noted that some distinctions were found in the bacterial communities between the different sample groups (surface and whole-body). For example, *Acinetobacter* and *Enterococcus* were more likely to inhabit the surface of the house crickets, while bacteria from the genera *Akkermansia* and *Paludibacter* were more likely to be related to the interior. A higher abundance of *Coprococcus* and *Lactococcus* bacteria in a whole-body Jamaican cricket samples (JW) than on the surface (JS) also indicates inside distribution. *Acinetobacter* and *Enterococcus* can be transmitted by water, food, or contact and are associated with some infectious diseases. The presence of these bacteria signals poor substrate hygiene [58,59]. A high content of bacteria on the surface of the crickets could be removed by washing the raw material before further processing. As a result, the washing step should be included in the cricket processing scheme, not only to remove feed residues or frass, but also as a preliminary microbiological safety measure.

Some bacteria from *Lactococcus*, *Akkermansia*, and *Coprococcus* genera are receiving increasing attention for their ability to regulate the gut microbiota and improve host health [60,61]. *Akkermansia* is a human intestinal mucin-utilizing symbiont, capable of enriching host metabolic and immune response functioning and is considered as a promising probiotic [60,62]. Butyric acid-producing *Coprococcus* bacteria are abundant in the human gut and their anti-inflammatory, neuroactive potential has been demonstrated [63]. Abundant in the intestinal tract or distributed in a wide range of fermented foods, representatives of *Paludibacter* and *Lactococcus* are responsible for polysaccharide fermentation, involved in the biochemical conversion of milk components [61,64,65]. The species of the latter genus are well known for their ability to produce lactic acid and its probiotic features [65]. *Trabulsiella* and *Lactococcus* have been listed as intestinal symbionts of phytophagous termites and are involved in the biodegradation of plant biomass, thus helping the host to digest and utilize its food [66,67,68]. Given that crickets are omnivorous and that plant-based foods are part of their natural diet, it can be assumed that orthopterans may also benefit from the symbiotic polysaccharide-fermenting bacteria that inhabit them, as is the case with termites or other animals. *Lactococcus* may also be valuable in the development of technologies to produce foods containing cricket ingredients. *Lactococcus garvieae* is known for its interaction with crickets and their growing environment [5]. Regarding that, this species was tested for abilities of spontaneous fermentation for cricket-wheat bakery production [40]. *Candidatus Azobacteroides* are intracellular symbionts of intestinal protists. They are nitrogen fixers and cellulose decomposers [69]. This bacterial group is associated only with insects and is mostly widespread in termites [70,71,72] but can also be found in cockroaches [73]. It is likely, crickets, as plant consumers, can benefit from these bacteria in similar ways as termites do.

The suitability of insects for human consumption cannot be judged only based on the microbiological composition of the unprocessed raw insect material [19,20]. Insect processing and storage conditions have a significant effect on the presence of foodborne pathogens [5,19,20,21]. Enterobacteriaceae (also detected during this study) is one of the main bacterial families related to hygiene and quality of the food [74]. Drying of insects, as single processing step, is not sufficient to inactivate most of Enterobacteriaceae and should be performed after boiling, which is more effective against them [75]. Our data are in line with others, demonstrating the effectiveness of the short boiling step in the removal of microbial contamination from analyzed crickets. It is worth noting that insufficient heat treatment without the elimination of spore-forming bacteria can lead to their rapid multiplication and the production of hazardous toxins, as there will be no other competitive bacteria left [21]. In our study, spore-forming bacteria from the *Bacillus* genus were isolated in different processing steps of crickets and their presence was observed in feeding substrate. Some *Bacillus* species, such as *B. cereus*, are included in the list of biological hazards of edible insects [20], while others, such as *B. flexus*, *B. subtilis*, etc. are known microbial symbionts of insects, usually non-pathogenic to humans [76,77]. Even more, *Bacillus spp*., due to the production of antimicrobial compounds, vitamins, carotenoids, etc. as well as lasting stability in processing chain and in the gastrointestinal environment, are gaining interest in functional food production and human health [78,79]. Numerous *Staphylococcus* genus bacteria (e.g., *S. epidermidis*, *S. warneri*, *S. hominis*) were isolated from processed *A. domesticus* and *G. assimilis* crickets. These species are not only abundant on human skin but were isolated from the gut of insects and could be related to the transfer of multidrug resistance [80]. Overall, more research is needed to formulate the most efficient raw cricket production steps and to elucidate potential microbiological risks. On the other hand, edible insect-associated microorganisms should gain much attention considering their beneficial, health-promoting features.

## 5. Conclusions

During this study, the bacterial community associated with Jamaican field cricket was characterized for the first time by applying the NGS approach and compared to a house cricket-inhabiting bacterial population. Analysis of the alpha and beta diversity of the bacterial communities, investigation of the distribution of microbial ASVs showed clear separation between bacteria inhabiting *A. domesticus* and *G. assimilis*. The core microbiomes forming *Parabacteroides* and *Bacteroides* dominated in both crickets tested, while the distribution pattern of unique ASVs from these genera varied in different cricket species and samples. Bacterial genera occurring at low frequency caused major differences in the structure of the microbiota. *Lactococcus*, *Candidatus Azobacteroides*, and *Coprococcus* prevailed in Jamaican field crickets, while *Enterococcus*, *Akkermansia*, and *Acinetobacter* dominated in house crickets. The efficiency of cricket processing was evaluated, and the high effectiveness of thermal treatment (boiling and oven-drying) was demonstrated in the removal of microbial contamination. Among the microorganisms, inhabiting both species of untreated crickets (as revealed by NGS analysis), as well as identified viable bacteria, surviving cricket processing, the potentially pathogenic bacteria were observed. These bacteria must be considered for possible biological hazards of cricket-based food. Nevertheless, among established prokaryotic microorganisms, natural inhabitants of the gastrointestinal tract of animals and humans, potentially beneficial probiotics were documented. These bacteria may be of great interest for functional food production and human health. The findings of this study will be helpful to culture both cricket species in controlled environments with proper antimicrobial feed ingredients that will increase the number of beneficial microbes and eliminate the microbial communities having detrimental properties. The obtained data will foster the development of strategies for cricket-based safe food production as well as the exploitation of beneficial properties of cricket-associated microorganisms.

## Figures and Tables

**Table 1:** The evaluation of 16S rRNA sequences in Jamaican (J) and house (H) crickets both surface (S) and whole-body (W) samples.

Samples	Reads Obtained	High Quality Reads	ASVs	Shannon’s Entropy	Faith’s PD
JS1	206,102	51,061	205	5.90	20.10
JS2	194,318	51,283	226	5.98	22.45
JS3	224,948	55,243	205	5.91	20.78
JW1	199,648	45,777	190	5.77	17.73
JW2	194,848	43,851	213	6.19	21.93
JW3	181,722	41,186	208	6.14	22.46
HS1	190,144	47,757	197	5.04	20.20
HS2	203,110	51,460	204	5.84	21.42
HS3	185,310	40,950	213	5.34	22.54
HW1	179,088	54,781	223	5.68	23.47
HW2	183,930	54,392	212	5.57	20.83
HW3	211,512	64,068	231	5.98	23.77
Total	2,354,680	601,809	2527		

**Table 2:** Beta diversity analysis based on both weighted and unweighted UniFrac distance metrics. JS—Jamaican cricket surface, JW—Jamaican cricket whole body, HS—house cricket surface, HW—house cricket whole-body samples.

	Unweighted Pseudo-F	UniFrac *p*-Value	Weighted Pseudo-F	UniFrac *p*-Value
JS vs. JW	1.870	0.095	2.531	0.11
JS vs. HS	5.209	0.116	23.038	0.102
JS vs. HW	3.327	0.102	10.394	0.104
JW vs. HS	3.349	0.086	2.370	0.184
JW vs. HW	3.116	0.105	1.946	0.214
HS vs. HW	2.018	0.101	3.972	0.208
Jamaican vs. house crickets	4.443	0.004	4.229	0.018

## Data Availability

Not applicable.

## Supplementary Materials

The following supporting information is available online at https://www.mdpi.com/article/10.3390/foods11081073/s1, Figure S1: Rarefaction curves for each sample, Figure S2: Relative abundance of bacterial taxonomy at the phylum (A) and family (B) levels for samples of Jamaican field (J) and house (H) crickets, Table S1: Bacterial taxonomy abundance count of Jamaican (J) and house (H) cricket samples, Table S2: Bacteria isolates detected in this study.
