# Supplementary figures and images for: The Bacterial Microbiota of Edible Insects Acheta domesticus and Gryllus assimilis Revealed by High Content Analysis
Source: Foods. 2022 Apr 7;11(8):1073. doi: 10.3390/foods11081073 (PMC9032608; doi:10.3390/foods11081073)

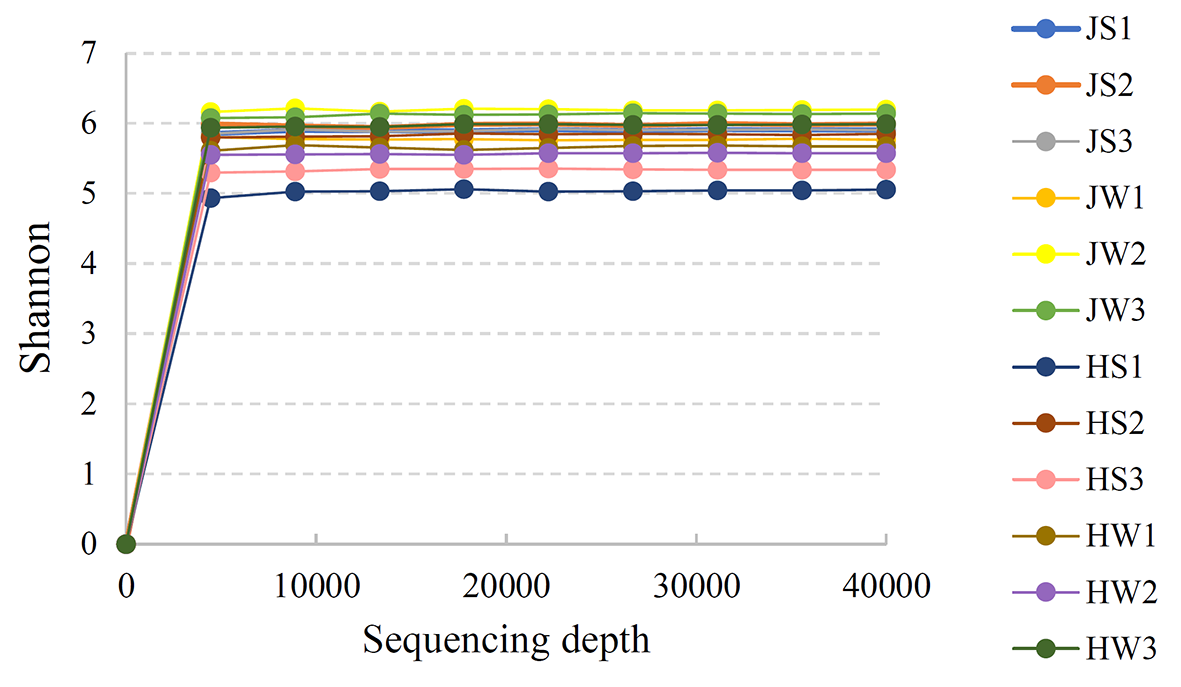

Supplement: Supplementary file 1 [file foods-11-01073-s001.zip › Figure S1.tif]

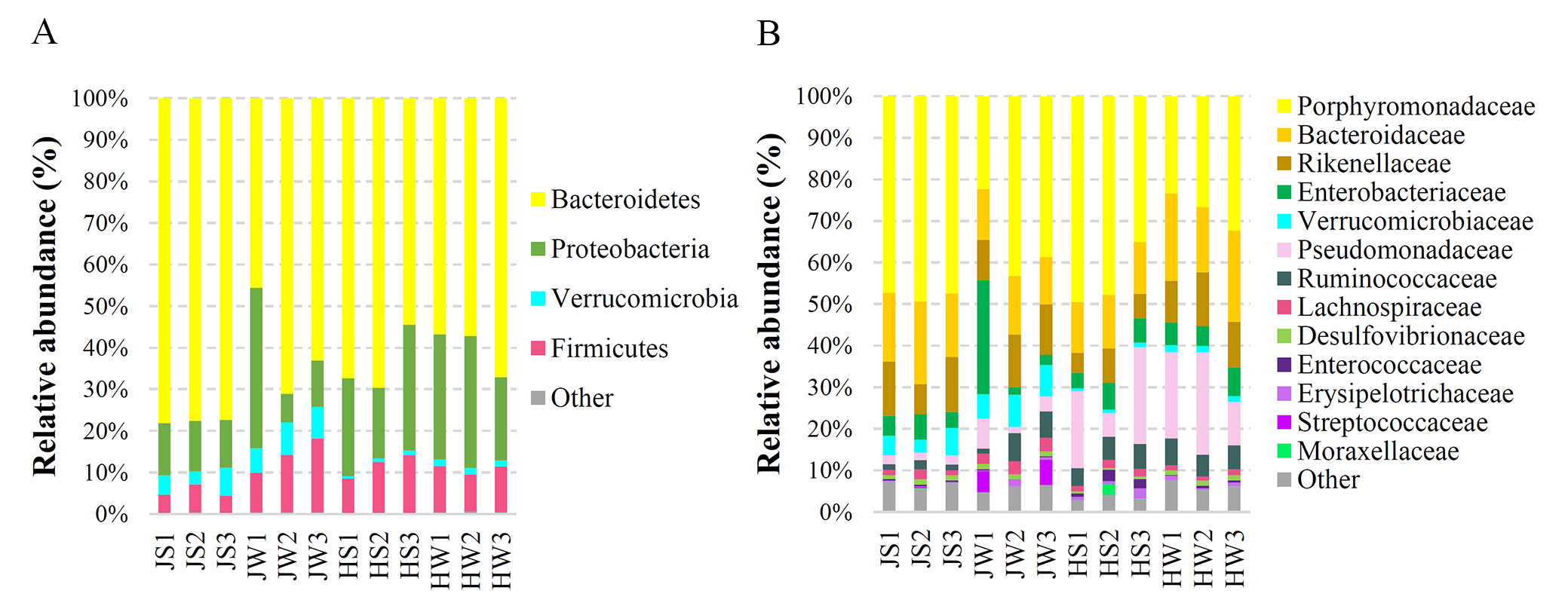

Supplement: Supplementary file 1 [file foods-11-01073-s001.zip › Figure S2.tif]
